# Characterization of the antibody response against EV71 capsid proteins in Chinese individuals by NEIBM-ELISA

**DOI:** 10.1038/srep10636

**Published:** 2015-05-29

**Authors:** Yingying Ding, Xuguang Chen, Baohua Qian, Guorong Wu, Ting He, Jiaojiao Feng, Caixia Gao, Lili Wang, Jinhong Wang, Xiangyu Li, Mingmei Cao, Heng Peng, Chunyan Zhao, Wei Pan

**Affiliations:** 1 Department of Medical Microbiology and Parasitology, School of Basic Medicine, Second Military Medical University; 2 Department of pediatrics, Wuxi people’s hospital, Jiangsu, China; 3 Department of Blood Transfusion, Changhai Hospital, Second Military Medical University, Shanghai, China; 4 Department of Clinical Laboratory, Wuxi people’s hospital, Jiangsu, China

## Abstract

Human enterovirus 71 (EV71) has become the major pathogen of hand, foot, and mouth disease (HFMD) worldwide, while the anti-EV71 antibody responses other than neutralizing epitopes have not been characterized. In this study, EV71 capsid proteins VP1, VP3, VP0 and various VP1 antigens were constructed to analyze anti-EV71 response in severe HFMD cases, non-HFMD outpatient children and normal adults using a novel evolved immunoglobulin-binding molecule (NEIBM)-based ELISA. The high prevalence of antibody responses against all three capsid proteins was demonstrated, and anti-EV71 VP1 showed the main antibody response. Anti-EV71 VP1 antibody response was found to predominantly target to epitopes based on the common enterovirus cross-reactive sequence. Moreover, inhibition pattern against anti-EV71 VP1 reactions in three groups was obviously different. Taken together, these results firstly characterized the anti-EV71 antibody responses which are predominantly against VP1 epitopes based on common enterovirus cross-reactive sequence. This finding could be helpful for the better understanding of anti-EV71 humoral immunity and useful for seroepidemiological surveillance.

As a small, non-enveloped, positive-stranded RNA virus with a genome approximately 7,400 bases in length, enterovirus 71 (EV71) is a member of the genus Enterovirus in the family Picornaviridae, which includes poliovirus, human enterovirus A, B, C and D (HEV-A–D), and more recently, human rhinovirus A, B and C, which infect humans. Along with human coxsackievirus (A2-8, A10, A12, A14 and A16), EV71 is classified into the species human enterovirus A, which is comprised of most causative agents responsible for hand, foot, and mouth disease (HFMD), based on its genome sequence^1^,^2^.

EV71 and CA16 are major etiological agents for HFMD, an exanthematous and self-limited febrile disease. However, a small proportion of EV71 acute infections have been associated with fatal neurological symptoms, including brain stem encephalitis, aseptic meningitis, and poliomyelitis-like paralysis^3^,^4^,^5^. First identified in California in 1969^6^, EV71 infection has been reported worldwide^7^,^8^,^9^. Several large outbreaks of HFMD, associated with EV71 infections, have been reported in Eastern and Southeastern Asian countries and regions during the late 20th century^8^,^9^,^10^,^11^. In China, large-scale outbreaks of HFMD associated with EV71 emerged in 2007, and nationwide epidemics have since continued and had become persistent^5^,^12^,^13^,^14^,^15^,^16^.

The host’s innate and adaptive immune responses play key roles in the infection and pathophysiology of viral infections. Studies concerning the host humoral immune responses against EV71 are primarily based on the neutralizing antibody assay. Approximately half of the neonates (50-57.6%) obtain protective neutralizing antibodies from their mothers; while as many as 90–98.0% infants lose neutralizing antibodies within 6-7 months, becoming vulnerable to EV71 infection^16^,^17^,^18^,^19^,^20^. Eventually, the accumulated seroprevalence of anti-EV71 neutralizing antibody reaches a peak level (above 80-100%) in children from 1 to 6 years of age, indicating that most primary infections were acquired during early childhood, and the adult group maintains a high seroprevalence of neutralizing antibody (40-85.3%) with a low incidence of HFMD^15^,^18^,^19^,^20^,^21^,^22^.

EV71 comprises 60 copies of four capsid proteins (VP1, VP2, VP3 and VP4) that form a symmetrical icosahedral structure. The capsid proteins VP1, VP2 and VP3 are exposed on the virus surface, and the smallest protein VP4 is arranged inside the icosahedral lattice^23^,^24^,^25^. The VP1 protein is highly exposed and has been suggested to play an important role in viral pathogenesis and virulence^26^,^27^,^28^. The viral structural proteins VP1, VP2 and VP3 all have beta-sandwich “jelly-roll” folds, and could be the principle targets for the host’s humoral immunity responses^23^,^29^,^30^,^31^,^32^. The neutralizing epitopes in the capsid have been identified^33^,^34^,^35^,^36^,^37^,^38^,^39^, but they only covered small part of exposed capsid, and couldn’t be all targets of anti-EV71 antibody responses. Whether there are the potential antibody responses other than the neutralizing antibody response and what they are remain unknown. In this study, we expressed the EV71 capsid proteins and a series of truncated VP1 proteins to systematically analyze the host antibody response to these proteins and demonstrated that human anti-EV71 antibody responses are predominantly activated in response to VP1, particularly to epitopes based on the common enterovirus cross-reactive sequence. This finding might contribute to a better understanding of anti-EV71 immunity and infection and could be useful for seroepidemiological surveillance and vaccine development.

## Results

### Production of recombinant EV71 capsid and truncated VP1 proteins

**Figure 1:**
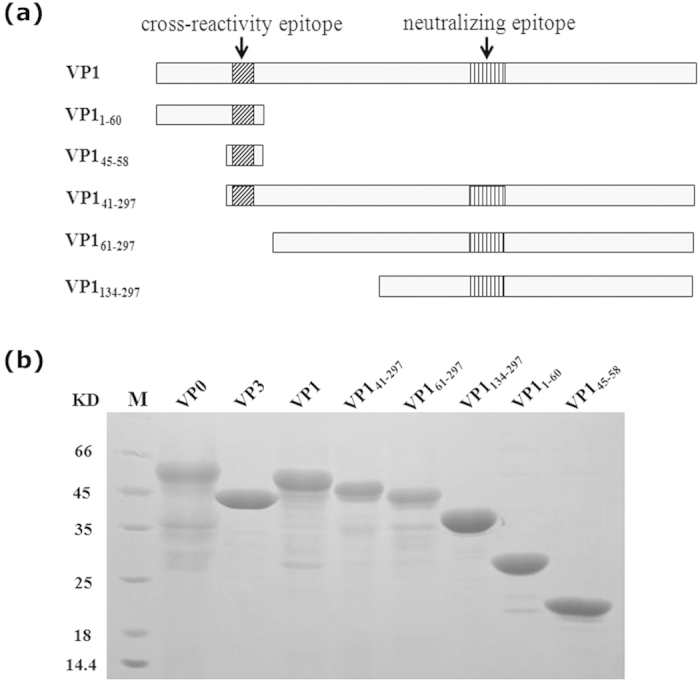
Design, expression and purification of full-length VP1 and truncated VP1 proteins. (**a**) The design of the five truncated VP1 proteins. (**b**) Expression and purification of recombinant full-length EV71 capsid proteins, VP0, VP1, and VP3 and the five truncated VP1 proteins. The expression of the recombinant full-length EV71 capsid proteins and truncated VP1 proteins was induced with IPTG, and the relative molecular weights (MW) of the expressed products were 52,898 for VP0, 44,151 for VP3, 50,371 for VP1, 46,230 for VP1_41-297_, 44,361 for VP1_61-297_ 36,111 for VP1_134-297_ and 23,730 for VP1_1-60,_ 19,002 for VP1_45-58_. The expressed products were purified using a Ni-NTA column affinity chromatography.

In addition to the three EV71 capsid proteins, VP0, VP1 and VP3, we also designed five truncated VP1 proteins, EV71 VP1_41-297_, VP1_61-297_, VP1_1-60_, VP1_134-297_ and VP1_45-58_ (Fig. 1a). These proteins were expressed in *E. coli* and investigated by SDS-PAGE (Fig. 1b) and the size of each recombinant protein was in agreement with the expected molecular weight. Some his-tag fusion proteins (EV71 VP0, VP3, VP1, VP1_41-297_, VP1_61-297_, VP1_134-297_) were found in inclusion bodies. Although these proteins were insoluble, they could be easily solubilized in 8 M urea and conveniently purified under denaturing conditions. The remaining proteins (VP1_1-60_, VP1_45-58_) were completely soluble. These soluble proteins were purified under native conditions.

### Establishment of anti-EV71 ELISA based on the novel evolved immunoglobulin-binding molecule (NEIBM)

**Figure 2:**
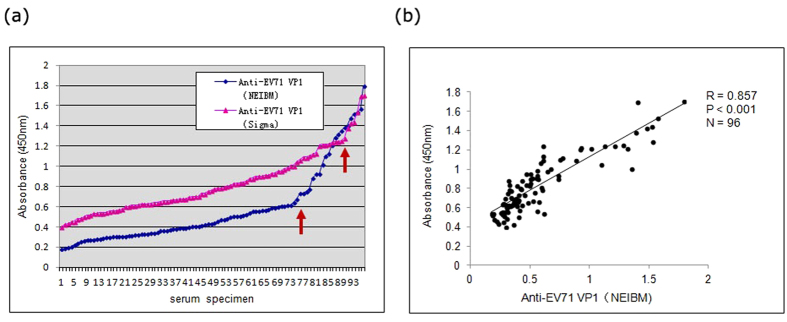
(**a**) Comparison of the anti-EV71 VP1 detection effect in 96 serum samples of normal adults using two different assays with commercial anti-human Igs conjugate (triangle) and NEIBM derived conjugate, HRP-LD5 (diamond). The distance between the red arrows indicates the wider positive detecting spectrum of NEIBM-ELISA than conventional ELISA. (**b**) The correlation of anti-EV71 VP1 reactivity detected using commercial anti-human Igs conjugate and NEIBM derived conjugate. The correlation was assessed using Spearman correlation coefficient. Correlation coefficient values (R), P values and the sample sizes (N) are shown.

NEIBM-based ELISA had been used in anti-HCV detection^40^ and anti-Tat antibodies analysis in Chinese individuals infected with HIV-1^41^. In this study, we compared the anti-EV71 VP1 detection effect in 96 serum samples of normal adults with two different ELISA assays using HRP-conjugated goat anti-human polyclonal polyvalent immunoglobulin and the NEIBM reporter molecule HRP-LD5. As shown in Fig. 2a, the NEIBM-based assay exhibited an obviously improved detection effect with stronger reactions of relatively high OD value and more clear reactions of background with relatively low OD value, comparing to the ELISA using the commercial anti-human Igs conjugate. The detections between these two assays were highly correlated, with an R value of 0.857 (Fig. 2b). Therefore, we used the NEIBM-based assay for anti-EV71 detection in this study.

The NEIBM-based anti-EV71 ELISA assay was established and used to detect anti-EV71 VP0, VP3 and VP1 antibody levels in 35 serum samples from normal adults. As there are no authentic anti-EV71 negative serum samples, it was impossible to determine the cutoff value for these anti-EV71 ELISA detection assays using the negative mean value and multiple standard deviations (SD). However, the anti-EV71 VP1 samples with OD values below 0.300 did not react strongly with VP3 or VP0 (Fig. S1), the anti-EV71 VP0 samples with OD values below 0.286 did not react strongly with VP3 or always with VP1, and the anti-EV71 VP3 samples with OD values below 0.200 did not always react strongly with VP0 or VP1. Thus, the cutoff values for anti-EV71 VP1, VP3 and VP0 were determined to be 0.300, 0.200 and 0.286, respectively.

### The anti-EV71 capsid antibody response is highly prevalent in severe HFMD cases, normal adults and non-HFMD outpatient children, and predominantly against VP1

**Figure 3:**
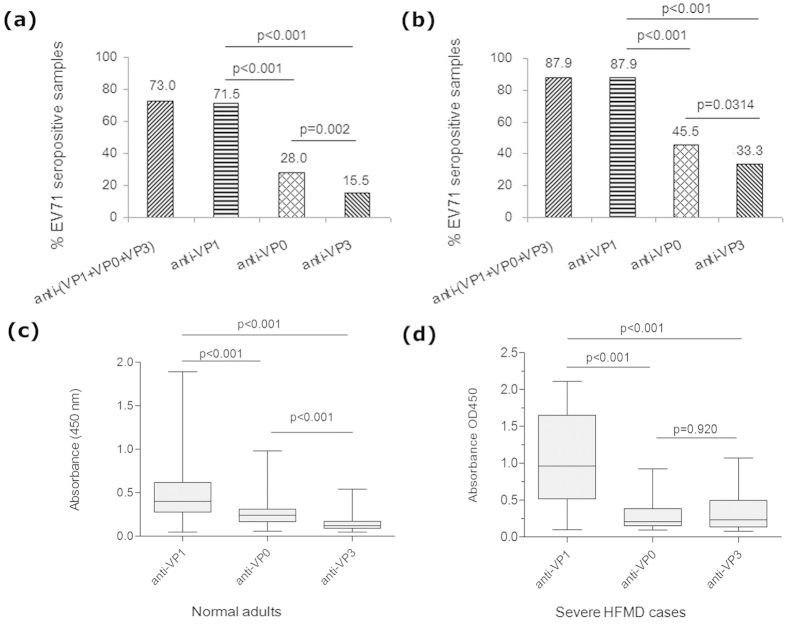
Characterization of the antibody responses to EV71 capsid proteins in 200 normal adults and 33 severe HFMD cases. The positive rate of antibody response was plotted on the y-axis against the three antigens plotted on the x-axis. The positive rate corresponding to the anti-(VP0 + VP1 + VP3) represented the total positive rate of antibody responses detected using EV71 VP0, VP1 and VP3 in 200 normal adults (**a**) and 33 severe HFMD cases (**b**). A comparison of the antibody reactivity against EV71 capsid VP0, VP3 and VP1 in 200 normal adults (**c**) and 33 severe HFMD cases (**d**). The boxes represent the interquartile range, the line inside each box represents the median of the samples and the whiskers represent the range of the data.

Using the NEIBM-based ELISA assay, we detected the anti-EV71 VP1, VP0 and VP3 antibody levels in serum samples of 200 normal adults and 33 severe HFMD cases. The high seroprevalence of anti-EV71 capsid proteins (VP1, VP0 and VP3) was found in both normal adults and severe HFMD cases, which were 73.0% and 87.9% respectively (Figs. 3a,b). To our surprise, the seroprevalence of anti-EV71 VP1 was almost as high as anti-EV71 capsid proteins, which were 71.5% and 87.9% in normal adults and severe HFMD cases respectively, significantly higher than that of anti-EV71 VP0 and VP3 in both normal adults (28.0% and 15.5%, respectively) and severe HFMD cases (45.5% and 33.3%, respectively). Consistently, the reactivity of anti-EV71 VP1 was also significantly stronger than that of anti-EV71 VP0 and VP3 in normal adults and severe HFMD cases, respectively (Figs. 3c,d). This result indicated that the anti-EV71 capsid antibody response is predominately stimulated against the VP1 protein.

### The common enterovirus cross-reactive sequence plays an important role in anti-EV71 VP1 antibody response

**Figure 4:**
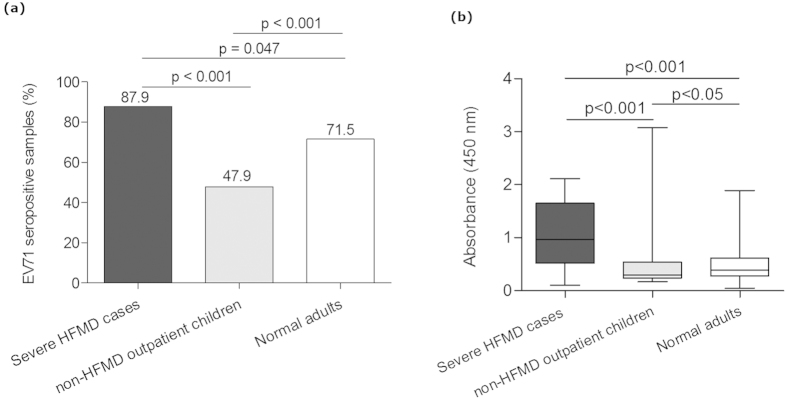
Characterization of the antibody responses to EV71 capsid VP1 in 33 severe HFMD cases, 200 normal adults and 194 non-HFMD outpatient children. (**a**) The positive rate of anti-VP1 antibody response was plotted on the y-axis against the three antigens plotted on the x-axis. (**b**) A comparison of the antibody reactivity against EV71 capsid VP1 in three groups. The boxes represent the interquartile range, the line inside each box represents the median of the samples and the whiskers represent the range of the data.

**Figure 5:**
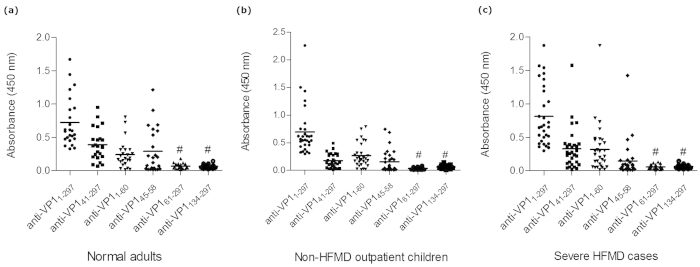
Comparison of the antibody reactivity against full-length and truncated VP1 proteins, EV71 VP1_41-297_, VP1_1-60_, VP1_45-58_, VP1_61-297_ and VP1_134-297_ in 24, 30 and 30 strongly VP1 reactive samples from normal adults (**a**), non-HFMD children (**b**) and severe HFMD cases (**c**), respectively. The boxes represent the interquartile range, the line inside each box represents the median of the samples and the whiskers represent the range of the data. # p < 0.001 comparing VP1_1-297_, VP1_41-297_, VP1_1-60_ or VP1_45-58_.

**Figure 6:**
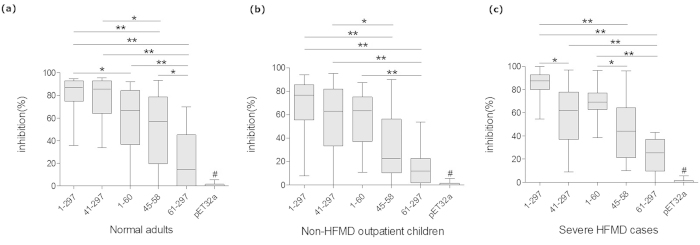
Comparison of inhibition activities of six proteins in normal adults (**a**), non-HFMD children (**b**) and severe HFMD cases (**c**). The percent inhibition of competitive ELISA was plotted on the y-axis with six inhibitor proteins on the x-axis: EV71 VP1, VP1_41-297_, VP1_1-60_, VP1_45-58_, VP1_61-297_ and pET32a. The data on the y-axis represent the inhibition of six proteins to coating antigens described in the “Methods” section. The boxes represent the interquartile range, the line inside each box represents the median of the samples and the whiskers represent the range of the data. Statistical significance was tested using Nemenyi non-parametric test. * represent p < 0.05, ** represent p < 0.001. # p < 0.001 comparing the five inhibitor proteins.

Anti-EV71 VP1 was also detected in 47.9% of non-HFMD outpatient children. Consistent with the results for seroprevalence, the anti-EV71 VP1 reactivity and positive rate in severe HFMD cases was significantly higher than that in normal adults and non-HFMD outpatient children (Fig. 4). To further define the principal reactive epitopes of VP1, a series of truncated VP1 proteins, VP1_41-297_, VP1_61-297_, VP1_1-60_, VP1_134-297_ and VP1_45-58_, were analyzed for the reactivity with 24, 30 and 30 VP1 strongly reactive samples (the serum samples were sorted by the OD value of anti-EV71 VP1 and the ones with highest reactivities were chosen to perform the indirect ELISA) from normal adults, non-HFMD outpatient children and severe HFMD cases, respectively. VP1_45-58_, VP1_41-297_ and VP1_1-60_ showed obvious reactivity with the VP1 strongly reactive samples, while VP1_61-297_ and VP1_134-297_ did not show obvious reactivity (Fig. 5). Interestingly, all reactive truncated VP1 proteins contain the common enterovirus cross-reactive sequence (VP1 45-58 aa), and the non-reactive proteins did not contain this sequence. Consistently, VP1_41-297_, VP1_1-60_, and VP1_45-58_, also exhibited significantly higher level of inhibition than VP1_61-297_ lacking of common enterovirus cross-reactivity sequence (Fig. 6). These results demonstrated that the common enterovirus cross-reactive sequence constitutes the essential component of the principal VP1 reactive epitopes and played an important role in anti-EV71 VP1 responses.

### The anti-EV71 VP1 in normal adults, non-HFMD outpatient children and severe HFMD exhibited the different characteristics

In order to further characterize the antigenicity of full length VP1 and various truncated VP1 proteins, 28, 28 and 27 VP1 strongly reactive samples (the serum samples were sorted by the OD value of anti-EV71 VP1 and the ones with highest reactivities were chosen) from 200 normal adults, 194 non-HFMD outpatient children and 33 severe HFMD cases were chosen to perform the competitive ELISA. The VP1 and truncated VP1 proteins containing common enterovirus cross-reactivity sequence, VP1_41-297_, VP1_1-60_, and VP1_45-58_, exhibited significantly higher level of inhibition than VP1_61-297_ lacking of common enterovirus cross-reactivity sequence in three groups, presenting a common characteristic of inhibition (Fig. 6). In contrast, VP1_1-60_ exhibited higher level of inhibition than VP1_45-58_ in severe HFMD cases, VP1_41-297_ exhibited higher level of inhibition than VP1_45-58_ in normal adults and non-HFMD outpatient children (Fig. 6), which could suggest the different anti-EV71 VP1 responses in severe HFMD cases, normal adults and non-HFMD outpatient children.

## Discussion

Anti-EV71 neutralizing antibody detection is currently the only assay to evaluate the seroprevalence of EV71 infection; however, this analysis does not provide detailed information concerning the characterization of the host antibody response to EV71 infection. Few studies have reported the use of the anti-EV71 ELISA assay because of its poor specificity and sensitivity. In the present study, we established a NEIBM-based ELISA assay and evaluated the antibody response of various anti-EV71 capsid proteins in severe HFMD cases predominantly infected by EV71 and normal adults as the control group originally to improve anti-EV71 ELISA detection efficacy for the diagnostic purpose. The NEIBM-based ELISA assay exhibited more numbers of detections with relatively high OD values and more clear detection background with relatively low OD values (Fig. 2), and showed obviously improved detection efficacy. The conjugates, NEIBM LD5, which synergistically binds to the VH3 and Vk regions of the Fab fragment of Igs, could enhance the IgM detection and contribute to the improved detection efficacy, while its well-defined binding sites and strict conformation-dependent binding mode might contribute to the improved specificity^42^. There has been no standard protocol for anti-EV71 ELISA detection yet because of some unresolved problems such as poor specificity, difficulty in cutoff value determination, etc. Theoretically, there are no authentic anti-EV71 negative groups; thus, the cutoff value for anti-EV71 ELISA detection could not be determined using typical negative mean values and multiple standard deviations (SD). We observed that the antigenicity of VP1, VP3 and VP0 was obviously different, and these differences provided the criteria to determine the cutoff values for anti-EV71 VP1, VP3 and VP0 detection in the present study (Fig. S1). Although the rationality should be further clarified, the detection results were reasonable. Using this assay, the seroprevalence of anti-EV71 VP1 was 71.5%, 47.9% and 87.9% in normal adults, non-HFMD outpatient children cases and severe HFMD cases, respectively, consistent with the reported neutralizing antibody seroprevalence rates of 50-83.3%, 28-58% and >80% in adults, outpatient cases and severe HFMD cases, respectively^13^,^43^,^44^,^45^. In addition, 64.55% of anti-EV71 VP1 detected using Western Blot analysis was reported in 141 sera samples collected from adults for regular health checkups and in 48 sera samples obtained from children without acute EV infections^46^.

In our study, anti-VP1 was found to be the predominant antibody response of anti-EV71 capsid proteins with the higher prevalence and stronger reactivity than anti-VP3 and VP0 in both severe HFMD cases and normal adults (Fig. 3). Whether our findings has any correlation with the fact that VP1 protein is highly exposed in virus surface and plays an important role in viral pathogenesis and virulence^24^,^25^,^47^,^48^ remain interesting. Moreover, the common enterovirus cross-reactive sequence plays the critical role in the anti-EV71 responses. Only proteins containing the common enterovirus cross-reactive sequence, full-length VP1, VP1_45-58_, VP1_41-297_ and VP1_1-60_, exhibited the strong antibody reactions, on the contrary, the VP1 proteins without this sequence, VP1_61-297_ and VP1_134-297_, did not exhibit strong reactions (Fig. 5). Consistently, VP1 proteins containing the common enterovirus cross-reactive sequence presented significantly stronger inhibitions to anti-VP1 than the protein (VP1_61-297_) without this sequence (Fig. 6). The common enterovirus cross-reactive sequence PALTA*ETG (* represents the variable or nonessential residues) was first characterized in coxsackievirus, echovirus, poliovirus, and enterovirus 70 in 1994 and was found to be the epitope responsible for the antibody cross-reaction between related enterovirus infections^43^. The equivalent sequence of EV71 VP1 (45-53 aa), PALTAVEIG, is also highly conserved among all causative agents of HFMD, including EV71, CA16, CA5, A10, A6, A19, CB3, Echo6, Echo4, coxsackievirus A4–A7, B2–B5, and enterovirus 18 (Table 1)^13^,^43^,^44^,^45^,^49^,^50^. Our results firstly revealed that common enterovirus cross-reactive sequence itself as well as its associated epitopes constitutes the key targets of anti-VP1 responses and stimulates the main anti-EV71 VP1 responses. We here proposed that VP1 epitopes based on the common enterovirus cross-reactive sequence could be classified into three kinds of antigens: core antigen, VP1_45-58_, containing one epitope or a few epitopes comprising of the common enterovirus cross-reactive sequence; N antigen, VP1_1-60_, containing epitopes comprising of both common enterovirus cross-reactive sequence and some amino acids in the N-terminal portion of VP1 at position of amino acid (aa) 1-44; C antigen, VP1_41-297_, containing epitopes comprising of both common enterovirus cross-reactive sequence and some amino acids in the region of 59-297 aa in the C-terminal portion of VP1. The full VP1 antigen consists of these three antigens and other epitopes without the common enterovirus cross-reactive sequence. Although the epitopes in VP1_45-58_ with only 14 amino acids in length should be much less than those in VP1_61-297_ with 237 amino acids in length, the antibody reactivity against VP1_45-58_ was at least equivalent to that against VP1_61-297_ in three groups, suggesting the predominant epitopes. Interestingly, the common enterovirus cross-reactive sequence is located in the interior of EV71 capsid and is not well exposed. Obviously, the predominant response to VP1 epitopes based on the common enterovirus cross-reactive sequence should not be stimulated by intact virus particles. The redundant VP1 produced during replication and some VP1 components degraded by endoproteinase might also play important role in antibody production. The nature of VP1 itself should play a crucial role in antibody induction. This finding revealed an immunological basis of cross reactions between anti-EV71 and anti-EV71 related viruses, and provided the potential mechanism for the poor specificity of conventional indirect anti-EV71 ELISA assay which was not suitable for diagnostic detection. Whether this cross reactions represent a common characteristic of anti-enterovirus humoral immunity and play the important role in anti-virus immunity remain interesting.

The inhibition patterns to anti-EV71 VP1 antibody response by various VP1 antigens showed obvious differences between three groups. In normal adults with mean age of 27 years who were most likely caused by the cross reactions from the recurrent or repeated infection with EV71-related viruses, VP1_41-297_ containing most sequence of VP1 exhibited almost identical inhibition to anti-EV71 VP1 compared to VP1_1-297_ and showed significantly stronger inhibition than VP1_45-58_ and VP1_1-60_ (Fig. 6). In contrast, in severe HFMD cases with mean age of 3.5 years who were predominantly infected with EV71 and had encountered the least infections with EV71-related viruses^14^, VP1_41-297_, VP1_1-60_ and VP1_45-58_ exhibited the lower level of inhibition than VP1_1-297_. Different with other two groups, VP1_1-60_ exhibited the relatively high level of inhibition compared to VP1_41-297_, and VP1_45-58_ in severe HFMD cases (Fig. 6). These results could reflect the characteristic of specific anti-EV71 reaction of primary EV71 infection. Consistently, the specific anti-EV71IgM detection was positive in 93.9% severe HFMD cases. In non-HFMD outpatient children with mean age of 3 years usually primarily infected with EV71 or EV71-related viruses with a relatively low seroprevalence compared to severe HFMD cases, the inhibition patterns seemed to be in the intermediate state between severe HFMD and normal adults, which might represent the combination of low prevalence of primary EV71 infection and/or primary infections with EV71-related viruses. Consistently, the specific anti-EV71IgM detection was positive in 22.5% non-HFMD outpatient children.

Neutralizing antibodies have been well studied and show high sensitivity and specificity. However, the antibody response characterized in the present study was different from that of neutralizing antibody. The major neutralizing epitope, SP70 (208-222 aa), was identified, and located in the C terminal of VP1^33^. In the present study, VP1_61-297_ and VP1_134-297_, which contain the neutralizing epitopes but not common enterovirus cross-reactive sequence showed significantly lower antigenicity than VP1_41-297_ in the ELISA assay. Consistent with our results, Zhang *et al.* also reported that His-VP1_49–297_, His-VP1_97–297_, His-VP1_148–297_, and His-VP1_202–297_ showed little reactivity with anti-EV71 rabbit, mouse and human sera and anti-VP1 mouse sera^51^. Our explanation for this discrepancy is that the EV71 infection induced very small amount of specific neutralizing antibodies which can only be detected by the sensitive neutralizing antibody assay but not ELISA assay. Consistently, the competitive ELISA demonstrated that VP1_61-297_ exhibited the significantly higher level of inhibition in comparison to the control protein, pET32a, but much lower level of inhibition in comparison to full-length VP1 and truncated VP1 proteins containing the common enterovirus cross-reactive sequence (Fig. 6). Based on these findings, we hypothesized the antibody response against VP1 epitopes based on common enterovirus cross-reactive sequence characterized in this study represents the major host antibody response to EV71 infection and could highly cross react with the EV71 related viruses, while the neutralizing antibody response represents the minor antibody response which shows high specificity and little cross reactivity. This finding should have some importance for vaccine development.

In conclusion, this study firstly characterized the host antibody responses against EV71 capsid proteins. The results demonstrated that human anti-EV71 antibodies are predominately to VP1, particularly to epitopes based on common enterovirus cross-reactive sequence. This finding might contribute to a better understanding of anti-EV71 immunity and infection and could be useful for seroepidemiological surveillance and vaccine development.

## Methods

### Ethics statement

Study protocol was approved by the Ethics Committee of Wuxi people’s hospital, Jiangsu, China. All experiments were performed in accordance with approved guidelines of the Ethics Committee of Wuxi people’s hospital and Second Military Medical University. Written informed consent was obtained from the participants in the study.

### Clinical samples

33 serum specimens of severe HFMD cases with the mean age of 3.5 years were collected within four days after onset from Wuxi people’s hospital, Jiangsu, China. Thirty one of thirty three serum samples (93.9%) were detected positive of IgM antibody against EV71 using an IgM μ-chain capture enzyme-linked immunoabsorbant assay (ELISA)^52^ (Beijing Wantai Biological Pharmacy Enterprise Co., Ltd., China). 194 clinical serum samples of non-HFMD children cases with the positive rate of 22.5% of specific anti-EV71IgM were also collected as the age-match controls from the Wuxi people’s hospital, Jiangsu, China. Informed consent was obtained from each of the participants prior to blood collection. Two hundred serum specimens were collected as normal adult controls from healthy blood donors at Changhai Hospital, Shanghai, China. The relevant information for each of the 427 samples was also recorded (Table S1). All samples were stored at −80 °C in 1.5 ml aliquots.

The diagnosis of HFMD was based on the clinical diagnostic guideline (2010) of the Ministry of Health of the People’s Republic of China and the specialist consensus on the clinical remedy of severe cases. The diagnostic criteria of severe cases of HFMD includes clinical diagnosis accompanied by one of the following symptoms: (1) long hyperpyrexia (not defervesce over three days); (2) myasthenia, convulsion, disturbance of consciousness, attenuation or disappearance of tendon reflex and positive meningeal irritation; (3) pale face, increased heart rate, poor peripheral circulation and dysarteriotony; (4) dyspnea or irregular rhythm and cyanosis; (5) increased peripheral blood leukocytes (>10 × 10^9^/L); and (6) an obvious increase in blood sugar (>9 mmol/L).

### Vectors, bacterial strain and reagents

The prokaryotic expression plasmid pET-32a and two *E. coli* host strains, BL21 (DE3) and Top10, were purchased from Novagen (Darmstadt, Germany). HRP-LD5 consists of HRP conjugated to LD5, which is a novel evolved immunoglobulin-binding molecule (NEIBM) with a characteristic structure of alternating B3 domain of Finegoldia magna protein L and D domain of staphylococcal protein A that creates synergistic double binding sites to the VH3 and Vk regions of Fab as well as to IgG Fc^42^. HRP-LD5 shows high binding affinity for IgM, IgG and IgA^40^. HRP-conjugated goat anti-human polyclonal polyvalent immunoglobulins (G, A, and M) (HRP goat anti-human PcAb) were obtained from Sigma (St. Louis, MO, USA).

### Clone of the EV71 capsid gene fragments and construction of the expression plasmids

The amino acid sequences of EV71-VP0, EV71-VP1 and EV71-VP3 (human enterovirus 71 capsid proteins VP0, VP1 and VP3) were obtained from GenBank (GenBank accession number EU703812, one of the Fuyang representative strain of HEV71: EV71/Fuyang.Anhui.P.R.C/17.08/1, C4a subgenotype strain of EV71). The encoded DNA sequences of EV71-VP0, EV71-VP1 and EV71-VP3 were synthesized using sequential OE-PCR^53^ and T/A-cloned into the pMD18-T vector (Takara). These constructs were used as templates to amplify VP0 and VP3 using the primer pairs uVP0/dVP0 and uVP3/dVP3 (Table S2), respectively. The primer pairs uVP1-0/dVP1-0, uVP1-1/dVP1-0, uVP1-2/dVP1-0, uVP1-4/dVP1-4, uVP1-5/dVP1-5 and uVP1-6/dVP1-6 (Table S2) were used to amplify VP1_1-297_, VP1_41-297_, VP1_61-297_, VP1_1-60_, VP1_134-297_ and VP1_45-58_ respectively, using the recombinant EV71-VP1 plasmid (VP1-pMD18-T) as a template. In addition, uVP0, uVP3, uVP1-0, uVP1-1, uVP1-2, uVP1-4, uVP1-5, and uVP1-6 contain BamHI restriction sites, and dVP0, dVP3, dVP1-0, dVP1-4, dVP1-5 and dVP1-6 contain HindIII restriction sites. The PCR products were inserted into the cloning sites (BamHI and Hind III) of the pET32a vector under the T7 promoter, and a His-tag was added at the N-terminus of the target to form a fusion protein. These expression plasmids were individually verified by sequencing analysis.

### Expression and purification of recombinant complete EV71 capsid and truncated VP1 proteins

*E. coli* BL21 (DE3) competent cells transformed with EV71 capsid proteins VP0, VP1, and VP3 and the five truncated VP1 expression plasmids were cultured in Luria broth (LB) medium supplemented with 100 μg/ml ampicillin (for *E. coli* transformed with pET32a vector) at 37 °C in a shaker at 200 rpm. When the OD_600_ of the culture reached 0.6, IPTG was added to a final concentration of 1 mM. After additional incubation for 2–3 h at 37 °C, the bacteria pellets were harvested through centrifugation at 6000 × g for 20 min, and the targeted proteins were detected using SDS-PAGE. The proteins were purified using Ni-NTA resin (Qiagen, Hilden, Germany). The purified proteins were immediately subpackaged and stored at −80 °C until further analysis^41^.

### Indirect ELISA of antibodies against EV71 capsid proteins

The anti-EV71 VP1, VP0 and VP3 ELISA detection using commercial anti-human Igs (immunoglobulins) conjugates and the NEIBM-derived conjugate HRP-LD5 (NEIBM-ELISA) were conducted as previously described^41^,^54^. Briefly, immunoassay strips (Nunc, Rochester, NY, USA) were coated with 1.0 μg of EV71-VP0, EV71-VP1, and EV71-VP3 in 50 mM carbonate buffer (pH 9.6) and incubated at 37 °C for 3 h. The strips were blocked for 2 h at 37 °C with 200 μl of 15% skimmed milk prepared in PBS-Tween 20. Next, 100 μl of a 20-fold dilution of the plasma sample was added to the appropriate wells. The strips were subsequently placed in a 37 °C incubator for 45 min. After washing four times with wash buffer (0.25% Tris base, 0.05% Tween 20), 100 μl of a 5,000-fold dilution of HRP-conjugated goat anti-human polyclonal polyvalent immunoglobulins (Sigma, St. Louis, MO, USA) or a 2,000-fold dilution of HRP-LD5 (1 mg/ml) was added to the strip and incubated for 45 min at 37 °C. The strips were developed using 3,3′,5,5′-tetramethylbenzidine (TMB) and hydrogen peroxide mixture. The reaction was stopped by the addition of 2 M sulphuric acid and the absorbance at 450 nm was read using an ELISA Reader (Biotek, Gene Company Limited, USA). The detections for anti-EV71 truncated VP1 proteins, VP1_41-297_, VP1_61-297_, VP1_1-60_, VP1_134-297_ and VP1_45-58_ were conducted as above.

### Competitive inhibition ELISA

To estimate the contribution of full length VP1 and various truncated VP1 proteins to the anti-EV71 VP1 reaction, the competitive inhibition ELISA was conducted as described^55^,^56^,^57^. Briefly, the 96-well microtiter plates was coated with 1.0 μg EV71 VP1 protein in 50 mM carbonate buffer (pH 9.6) overnight at 4 °C and then were blocked for 2 h at 37 °C with 200 μl of 15% skimmed milk prepared in PBS-Tween 20. 100 μl of 20-fold dilution of the plasma samples with high anti-VP1 antibody response from three groups first reacted with 2.0 μg inhibitor proteins, full-length VP1, VP1_41-297_, VP1_61-297_, VP1_1-60_, VP1_45-58_ and pET32a for 1 h at 37 °C respectively, then the serum in the presence (test serum) and absence (serum control) of inhibitor proteins were added into VP1-coated strips and incubated for 45 min at 37 °C. After incubation, the plates were washed four times with wash buffer followed by 100 μl of a 2,000-fold dilution of HRP-LD5 (1 mg/ml). Plates were incubated for 45 min at 37 °C and then washed. The plates were developed using 3,3′,5,5′-tetramethylbenzidine and hydrogen peroxide mixture. The reaction was stopped after suitable color development by the addition of 2 M sulphuric acid and the absorbance at 450 nm was read using an ELISA Reader. Three parallel wells of each test were detected, and the mean of the absorbance from three wells were used to calculate the percentage of inhibition. The percentage of inhibition (PI) was calculated as follows: PI = [100 − (absorbance value of test serum – absorbance value of background)/(absorbance value of serum control – absorbance value of background) × 100)], where absorbance of background was obtained in the absence of sample or HRP-LD5.

### Statistical analyses

Statistical analyses were performed using SPSS 17.0 and SAS 9.3 software. All experiments were performed in triplicate, and the values obtained from three replicate samples were averaged for each experiment. The statistical significance was tested using chi-square test and non-parametric test. Differences between measurements were considered significant at p-values less than 0.05.

## Additional Information

**How to cite this article**: Ding, Y. *et al.* Characterization of the antibody response against EV71 capsid proteins in Chinese individuals by NEIBM-ELISA. *Sci. Rep.* **5**, 10636; doi: 10.1038/srep10636 (2015).

## Supplementary Material

Supplementary Information

## Figures and Tables

**Table 1:** Alignment of amino acid sequences of common cross-reactivity epitope of VP1 (e.g. EV71 VP1_45-53_) from different enteroviruses.

**Virus**	**Species**	**Sequence**									**Reference**
CA5*	Human enterovirus A	P	A	L	Q	A	A	E	T	G	ACT52615.1**
CA6*	Human enterovirus A	-	-	-	-	-	-	-	-	-	44
CA10*	Human enterovirus A	-	-	-	-	-	-	-	-	-	ADF43062.1
CA16*	Human enterovirus A	-	-	-	-	-	-	-	-	-	13
EV71*	Human enterovirus A	-	-	-	-	-	-	-	I	-	13
CB3*	Human enterovirus B	-	-	-	T	-	-	-	-	-	AAD50446.1
Echo4*	Human enterovirus B	-	N	-	T	-	V	-	-	-	AAF36390.1
Echo6*	Human enterovirus B	-	-	-	T	-	-	-	-	-	AAA65044.1
HPV1	Human enterovirus C	-	-	-	T	-	V	-	-	-	43
HEV70	Human enterovirus D	-	S	--	N	-	V	-	-	-	45
HRV8	Human rhinovirus A	-	-	-	D	-	-	-	-	-	50
HRV26	Human rhinovirus B	-	-	-	T	-	N	-	-	-	49
HRVC	Human rhinovirus C	S	-	-	G	-	M	-	I	-	AEM44644.1

CA5:Human coxsackievirus A5, CA6:Human coxsackievirus A6, CA10:Human coxsackievirus A10, CA16:Human coxsackievirus A16, EV71:Human enterovirus 71, CB3:Human coxsackievirus B3, Echo4:Human echovirus 4, Echo6:Human echovirus 6; HPV1:Human poliovirus 1, HRV26:Human rhinovirus 26, HRV8:Human rhinovirus 8, HEV70:Human enterovirus 70, HRVC:Human rhinovirus C

The asterisks (*) represent causative agents of HFMD. The dash (-)indicates conserved amino acid. (**) indicates accession number of Genebank.
