# Supplementary material for: Characterization of the antibody response against EV71 capsid proteins in Chinese individuals by NEIBM-ELISA
Source: Sci Rep. 2015 May 29;5:10636. doi: 10.1038/srep10636 (PMC4448526; doi:10.1038/srep10636)
Supplement: Supplementary Information [file srep10636-s1.pdf]

Characterization of the antibody response against EV71 capsid proteins in Chinese individuals by NEIBM-ELISA

Yingying Ding[1], Xuguang Chen[2], Baohua Qian[3], Guorong Wu[4], Ting He[1], Jiaojiao Feng[1], Caixia Gao[1], Lili Wang[1], Jinhong Wang[1], Xiangyu Li[1], Mingmei Cao[1], Heng Peng[1], Chunyan Zhao[1], Wei Pan[1*]

[1]Department of Medical Microbiology and Parasitology, School of Basic Medicine, Second Military Medical University.

[2]Department of pediatrics, Wuxi people's hospital, Jiangsu, China.

[3]Department of Blood Transfusion, Changhai Hospital, Second Military Medical University, Shanghai, China,

[4]Department of Clinical Laboratory, Wuxi people's hospital, Jiangsu, China.

*Send correspondence to Prof. Wei Pan, Department of Medical Microbiology and Parasitology, School of Basic Medicine, Second Military Medical University, Shanghai 200433, China.

Tel: +86 21 81870989. E-mail: pwpanwei@126.com

(a)

NO.	anti-VP1	anti-VP0	anti-VP3	pET32a
59	1.834	0.255	0.103	0.094
42	1.61	0.28	0.097	0.06
187	1.477	0.282	0.158	0.064
97	1.321	0.295	0.107	0.089
131	1.282	0.278	0.171	0.076
177	1.1	0.289	0.261	0.077
51	1.076	0.255	0.077	0.092
163	0.926	0.287	0.147	0.089
172	0.774	0.279	0.186	0.097
49	0.747	0.264	0.11	0.052
122	0.674	0.287	0.338	0.098
3	0.669	0.272	0.133	0.032
67	0.618	0.304	0.1	0.065
118	0.608	0.301	0.189	0.096
191	0.598	0.281	0.139	0.074
153	0.591	0.282	0.265	0.068
65	0.589	0.269	0.125	0.099
136	0.553	0.286	0.322	0.08
178	0.534	0.296	0.209	0.085
165	0.524	0.3	0.166	0.09
170	0.451	0.278	0.156	0.072
40	0.434	0.263	0.109	0.085
193	0.433	0.269	0.12	0.068
92	0.423	0.278	0.101	0.082
162	0.393	0.286	0.184	0.07
200	0.391	0.288	0.139	0.056
142	0.379	0.279	0.17	0.066
90	0.368	0.302	0.163	0.045
126	0.313	0.294	0.262	0.064
50	0.306	0.264	0.156	0.081
12	0.283	0.276	0.104	0.064
34	0.274	0.281	0.089	0.098
88	0.243	0.28	0.133	0.056
39	0.219	0.278	0.11	0.078
66	0.204	0.28	0.118	0.064

(b)

NO.	anti-VP1	anti-VP0	anti-VP3
67	0.618	0.304	0.1
90	0.368	0.302	0.163
118	0.608	0.301	0.189
165	0.524	0.3	0.166
178	0.534	0.296	0.209
97	1.321	0.295	0.107
126	0.313	0.294	0.262
177	1.1	0.289	0.261
200	0.391	0.288	0.139
122	0.674	0.287	0.338
163	0.926	0.287	0.147
136	0.553	0.286	0.322
162	0.393	0.286	0.184
153	0.591	0.282	0.265
187	1.477	0.282	0.158
191	0.598	0.281	0.139
34	0.274	0.281	0.089
42	1.61	0.28	0.097
66	0.204	0.28	0.118
88	0.243	0.28	0.133
142	0.379	0.279	0.17
172	0.774	0.279	0.186
39	0.219	0.278	0.11
92	0.423	0.278	0.101
131	1.282	0.278	0.171
170	0.451	0.278	0.156
12	0.283	0.276	0.104
3	0.669	0.272	0.133
65	0.589	0.269	0.125
193	0.433	0.269	0.12
49	0.747	0.264	0.11
50	0.306	0.264	0.156
40	0.434	0.263	0.109
51	1.076	0.255	0.077
59	1.834	0.255	0.103

(c)

NO.	anti-VP1	anti-VP0	anti-VP3
122	0.674	0.287	0.338
136	0.553	0.286	0.322
153	0.591	0.282	0.265
126	0.313	0.294	0.262
177	1.1	0.289	0.261
178	0.534	0.296	0.209
118	0.608	0.301	0.189
172	0.774	0.279	0.186
162	0.393	0.286	0.184
131	1.282	0.278	0.171
142	0.379	0.279	0.17
165	0.524	0.3	0.166
90	0.368	0.302	0.163
187	1.477	0.282	0.158
170	0.451	0.278	0.156
50	0.306	0.264	0.156
163	0.926	0.287	0.147
200	0.391	0.288	0.139
191	0.598	0.281	0.139
88	0.243	0.28	0.133
3	0.669	0.272	0.133
65	0.589	0.269	0.125
193	0.433	0.269	0.12
66	0.204	0.28	0.118
39	0.219	0.278	0.11
49	0.747	0.264	0.11
40	0.434	0.263	0.109
97	1.321	0.295	0.107
12	0.283	0.276	0.104
59	1.834	0.255	0.103
92	0.423	0.278	0.101
67	0.618	0.304	0.1
42	1.61	0.28	0.097
34	0.274	0.281	0.089
51	1.076	0.255	0.077

Figure S1. Determination of the cutoff values for anti-EV71 ELISA detection in sera samples from normal adults. (a) The samples of anti-EV71 VP1 with OD values below 0.300 (tentative cutoff value) did not react overtly with VP3 or VP0; (b) the samples of anti-EV71 VP0 with OD values below 0.286 (tentative cutoff value) did not react overtly with VP3 and did not always react strongly with VP1; (c) the samples of anti-EV71 VP3 with OD values below 0.200 (tentative cutoff value) did not always react strongly with VP3 and VP1.

Table 1. Baseline characteristics of the study participants

	Blood Donor	non-HFMD Children	Severe HFMD cases
**Number**[a]	200	194	33
**Time**	2011.2.24-3.17	2011.6.27-7.4	2011.6.27-7.4
**Age (yr)**			
Mean ± s.d.[b]	27.0 ± 7.6	3.0 ± 2.4	3.5 ± 2.6
Range	18-48	1-12	1-11
**Sex (%)**			
Male	52.5%	59.3%	60.6%
Female	47.5%	40.7%	39.4%

[a]Number of evaluable individuals; [b]Standard deviation;

Table 2. Primers for amplifying EV71 capsids and truncated VP1.

Primers	Sequences (5'- 3')	Description
uVP0	GCGCGCGGATCCATGGGTTCTCAGGTTTCT	The primer pairs uVP0/dVP0 and uVP3/dVP3 were used to amplify VP0 and VP3, respectively. uVP0, uVP3 contain BamHI restriction sites (underlined), dVP0, dVP3 contain HindIII restriction site (underlined).
dVP0	GCGCGCAAGCTTTTACTGAGTAACAGCCTGACG	
uVP3	GCGCGCGGATCCGGTTTCCCGACTGAACTG	
dVP3	GCGCGCAAGCTTTTACTGGATAGTACCAGTCTG	
uVP1-0	GCGCCGGGATCCGGTGACCGTGTTGCTGAC	The primer pairs uVP1-0/dVP1-0, uVP1-1/dVP1-0 and uVP1-2/dVP1-0 were used to amplify VP1$_{1-297}$, VP1$_{41-297}$, VP1$_{61-297}$, respectively. uVP1-0, uVP1-1, uVP1-2 contain BamHI restriction sites (underlined), dVP1-0 contain HindIII restriction site (underlined).
uVP1-1	GCGCGCGGATCCACTGGTAAAGTTCCGGCTCTG	
uVP1-2	GCGCGCGGATCCGAATCTATGATCGAAACTC	
dVP1-0	GCGCCGAAGCTTCTACAGAGTAGTGATAGCAGT	
uVP1-4	CGCGCGGGATCCGGTGACCGTGTTGCTGACGT	The primer pairs uVP1-4/dVP1-4, uVP1-5/dVP1-5,and VP1-6/dVP1-6 were used to amplify VP1$_{1-60}$, VP1$_{134-297}$, and VP1$_{45-58}$, respectively. uVP1-4, uVP1-5, uVP1-6 contain BamHI restriction sites (underlined), dVP1-4, dVP1-5 and dVP1-6 contain HindIII restriction site (underlined).
dVP1-4	GCCGGCAAGCTTTTAGTCAGAAGCGTTAGAAGAAG	
uVP1-5	CGCGGCGGATCCGAATTCACTTTCGTTGCTTG	
dVP1-5	GCCGGCAAGCTTTTACAGAGTAGTGATAGCAGTACGAGA	
uVP1-6	CGCGCGGGATCCCCGGCTCTGCAGGCTGCTG	
dVP1-6	GCCGGCAAGCTTTTAAGCGTTAGAAGAAGCACC	
